# A mirror code for protein-cholesterol interactions in the two leaflets of biological membranes

**DOI:** 10.1038/srep21907

**Published:** 2016-02-26

**Authors:** Jacques Fantini, Coralie Di Scala, Luke S. Evans, Philip T. F. Williamson, Francisco J. Barrantes

**Affiliations:** 1 EA-4674, Interactions Moléculaires et Systèmes Membranaires, Aix-Marseille Université, Marseille, France; 2 Centre for Biological Sciences/Institute for Life Sciences, University of Southampton, Southampton, SO17 1BJ, United Kingdom; 3 Laboratory of Molecular Neurobiology, Biomedical Research Institute (BIOMED) UCA–CONICET, Faculty of Medical Sciences, Catholic University of Argentina, Buenos Aires, Argentina

## Abstract

Cholesterol controls the activity of a wide range of membrane receptors through specific interactions and identifying cholesterol recognition motifs is therefore critical for understanding signaling receptor function. The membrane-spanning domains of the paradigm neurotransmitter receptor for acetylcholine (AChR) display a series of cholesterol consensus domains (referred to as “CARC”). Here we use a combination of molecular modeling, lipid monolayer/mutational approaches and NMR spectroscopy to study the binding of cholesterol to a synthetic CARC peptide. The CARC-cholesterol interaction is of high affinity, lipid-specific, concentration-dependent, and sensitive to single-point mutations. The CARC motif is generally located in the outer membrane leaflet and its reverse sequence CRAC in the inner one. Their simultaneous presence within the same transmembrane domain obeys a “mirror code” controlling protein-cholesterol interactions in the outer and inner membrane leaflets. Deciphering this code enabled us to elaborate guidelines for the detection of cholesterol-binding motifs in any membrane protein. Several representative examples of neurotransmitter receptors and ABC transporters with the dual CARC/CRAC motifs are presented. The biological significance and potential clinical applications of the mirror code are discussed.

It is now well established that cholesterol controls the activity of a wide range of membrane receptors through general effects on fluidity^1^,^2^,^3^, membrane curvature^4^,^5^ or, more specifically, through direct binding to the membrane-spanning domains of these receptors^6^,^7^,^8^,^9^,^10^. The latter effect is particularly interesting since in this way cholesterol is able to control receptor conformation and dimerization, as shown for G-protein-coupled receptors^11^. Identifying cholesterol recognition motifs in the transmembrane (TM) domains of signaling receptors has therefore become a critical undertaking for understanding the molecular mechanisms controlling signaling functions^9^,^12^. In this respect, computational approaches have revealed interesting structural features pointing to the way in which proteins specifically interact with cholesterol^8^,^13^,^14^,^15^,^16^ and have led to the definition of consensus motifs that can be used as reliable algorithms for identifying cholesterol-binding domains. Though the cholesterol recognition amino acid consensus (CRAC) motif^13^, defined by the linear array (L/V)-X_1−5_-(Y)-X_1−5_-(K/R), is probably the most widely used sequence-based tool to predict the presence of a cholesterol-binding domain in a protein, its relevance may not extend to all proteins, as in the case of the nicotinic acetylcholine receptor (AChR)^8^. The search for alternative cholesterol binding sites in this receptor led us to identify a new consensus motif, referred to as the “CARC” domain^8^, which is a reverse version of the CRAC algorithm: (K/R)-X_1−5_-(Y/F)-X_1−5_-(L/V). CARC appears to be superior to CRAC in predicting the presence of cholesterol binding in the nicotinic receptor as well as in a series of other membrane proteins^9^. In fact, analysis of the energy of interaction of cholesterol with a collection of CRAC and CARC motifs revealed that a CARC domain generally exhibits more affinity for cholesterol than a CRAC domain^9^. The biochemical grounds for such a mode of interaction, including the snorkeling effect of Lys/Arg residues^17^ and cholesterol structure^12^, have been discussed in a recent review^9^. However, most of the data describing the modalities of CARC-cholesterol interactions were obtained in silico^8^,^9^. It therefore remained to be established whether cholesterol could physically interact with a CARC domain and if so, whether this interaction is of high affinity, lipid-specific and saturable. Moreover, although the central aromatic residue defined by the CARC algorithm appeared highly critical for cholesterol binding in modeling studies, the impact of mutations at this position was not proven experimentally. For these reasons, we decided to conduct a series of physicochemical experiments with a prototype CARC peptide to test the interaction of this peptide with cholesterol. The results of these studies are presented in the first part of this work. We also noticed that some TM domains display both CARC *and* CRAC domains, suggesting the intriguing possibility that these TM domains could bind two cholesterol molecules, one in each leaflet of the membrane. This unique feature was studied in a set of randomly selected TM domains belonging to a broad range of membrane proteins, including G-protein coupled receptors, voltage-gated channels, and the mitochondrial translocator protein TSPO. This search allowed us to decipher the “mirror” code controlling the dual association of cholesterol with TM domains in both the outer and inner membrane leaflets. Cracking this code enabled us to formulate guidelines for the accurate detection of cholesterol-binding motifs in membrane proteins.

## Results

### Selection of a prototype CARC motif in the AChR

**Figure 1:**
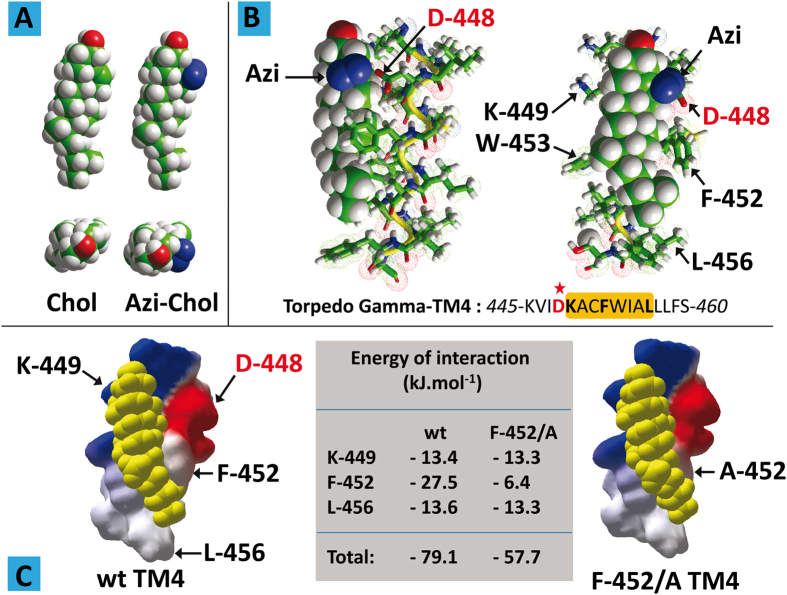
Molecular modeling studies of CARC-cholesterol interactions. (**A**) Front and top views of the molecular structure of cholesterol (Chol) and Azi-cholesterol (Azi-Chol). (**B**) Molecular modeling simulations of the interaction between Azi-cholesterol (in sphere rendition) and γ-TM4 (fragment 445–460 in tube rendition. The position of Asp-448 in the amino acid sequence of γ-TM4 is indicated by a red asterisk. The CARC domain containing Lys-449, Phe-452 and Leu-456 (bold characters) is framed in orange. (**C**) Docking of cholesterol (yellow spheres) on wild-type (wt) and mutant (F-452/A) of γ-TM4. The energy of interaction of each complex is indicated in the central table.

The 4^th^ transmembrane domain (TM4) of the human AChR γ subunit displays a typical CARC motif: 455-**R**VC**F**LAM**L**-462 (with the Arg, Phe, and Leu amino acid residues defined by the CARC algorithm bold and underlined). Molecular modeling simulations suggest that this domain displays a remarkable fit for cholesterol^8^, with a predicted energy of interaction of −60 kJ.mol^−1^. Interestingly, photolabeling studies using [^3^H]Azicholesterol (Fig. 1A) have identified this region as an actual cholesterol-binding domain in the *Torpedo* AChR^18^.

The *Torpedo* TM4 sequence of the AChR γ subunit displays a CARC motif similar to the human form, yet, as expected for a phylogenetically distant species, with some variations: 449-**K**AC**F**WIA**L**-456. Most importantly, the main [^3^H]Azicholesterol incorporation into *Torpedo* TM4 was Asp-448, which is the immediate N-terminal neighbor of the first amino acid (Lys-449) of the CARC motif. In order to understand how Asp-448 reacts with [^3^H]Azicholesterol, we performed a series of molecular dynamics simulations of the cholesterol derivative with a *Torpedo* γ-TM4 segment (445–460) encompassing the CARC domain together with a few upstream and downstream amino acid residues (Fig. 1B).

A remarkable fit between the TM4 domain and Azicholesterol was found, involving chiefly Lys-445, Phe-452, Trp-453, and Leu-456 residues. In this complex, the reactive Azi group of the cholesterol derivative was as close as 2 Å from the side chain of Asp-448. Indeed, the carboxylate group of Asp-448 was the atomic group closest to Azicholesterol. In particular, the lateral Azi-group did not interact with any of the apolar amino acid residues of TM4, in full agreement with the lack of radioactive labeling of these residues^18^. We studied next the interaction of natural cholesterol with the TM4 domain of *Torpedo* AChR γ-subunit (Fig. 1C). As was the case with the human receptor, we found a remarkable fit between cholesterol and the *Torpedo* AChR domain. The energy of interaction between cholesterol and *Torpedo* AChR γ-TM4 was close to −80 kJ.mol^−1^, which is the highest ever obtained^9^ for any protein-cholesterol complex. The amino acid triad defining the CARC algorithm (Lys-449/Phe-452/Leu -456) by itself accounted for 68.9% of the whole energy of interaction. The interaction between cholesterol and the central aromatic residue of CARC (Phe-452) was by far the most important energetic contributor (−27.5 kJ.mol^−1^). Correspondingly, replacing Phe-452 with alanine (F-452/A mutant) resulted in a significant loss of interaction, as shown in Fig. 1C. Finally one should note that Asp-448 was not involved in cholesterol binding, which is consistent with the lack of reactive Azi group in natural cholesterol. Nevertheless, the molecular modeling study suggests that the CARC domain of *Torpedo* AChR γ-TM4 could bind both natural cholesterol and Azi-cholesterol through similar interactions involving the main CARC amino acid residues.

**Figure 2:**
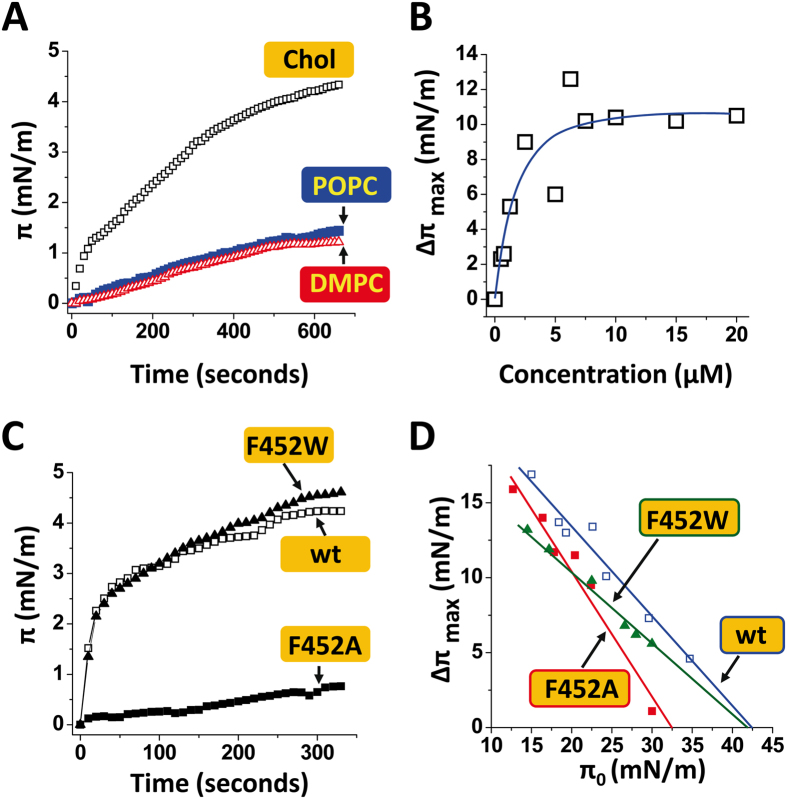
Physicochemical studies of CARC-cholesterol interactions. (**A**) Kinetics of interaction of a synthetic γ-TM4 peptide (fragment 445–460 of Torpedo AChR γ-subunit) with a monolayer of cholesterol (Chol) or phosphatidylcholine (palmitoyl-oleoyl phosphatidylcholine, POPC or dimyristoyl phosphatidylcholine, DMPC). (**B**) Effect of peptide concentration on the interaction between γ-TM4 and cholesterol (Δπ_max_ measured after 30 min of incubation). (**C**) Kinetics of interaction of wild-type (wt) and mutant peptides (F-452/A; F-452/W) derived from γ-TM4 with cholesterol monolayers (π_0_ = 30 ± 3.5 mN.m−1). **D.** Maximal surface pressure increase (Δπ_max_) induced by wild-type and mutant peptides on cholesterol monolayers prepared at various π_0_ values.

On the basis of these in silico data, we performed a series of physicochemical experiments to test whether cholesterol physically interacts with the CARC motif of *Torpedo* AChR γ-TM4. In these experiments, a synthetic peptide derived from this domain (445–460) was injected underneath a lipid monolayer prepared at the air-water interface. The interaction of the peptide with the reconstituted lipid membrane was quantitatively assayed by surface pressure measurements^19^. This technique has been validated as one of the most powerful and reliable approaches for studying protein-lipid interactions under well-controlled experimental conditions^19^,^20^,^21^. First we analyzed the interaction of the prototype CARC peptide with cholesterol (Fig. 2A). Immediately after the injection of the peptide, the surface pressure started to increase, indicating that the peptide in solution had a high affinity for the cholesterol-containing membrane. When the peptide was added onto a pure phosphatidylcholine monolayer (either POPC or DMPC), the surface increase was comparatively very small. These data demonstrate that the peptide had a higher affinity for cholesterol relative to phosphatidylcholine. Thus, the CARC motif exhibits specific cholesterol-binding properties, and not just a broad lipid-binding activity. Moreover, the interaction of CARC with cholesterol was saturable, with a maximal value in the 5–20 μM peptide concentration range (Fig. 2B).

Finally we studied the impact of single-point mutations on CARC-cholesterol interactions. Kinetic studies indicated that the F-452/A mutation (Phe-452 replaced by Ala) had a marked impact on the initial velocity of the interaction (Fig. 2C). In contrast, the F-452/W mutant (Phe-452 replaced by Trp) behaved as the wild-type peptide, indicating that the homologous aromatic/aromatic substitution did not significantly affect cholesterol binding. This observation was confirmed by comparing the interaction of the mutant and wild-type peptides with cholesterol monolayers prepared at various initial pressure (π_0_) values (Fig. 2D). These specific curves allowed us to determine the critical pressure of insertion (π_c_) which corresponds to the density of the monolayer at which the interaction no longer occurs, due to an excessive lipid concentration. At this point, lipid-lipid interactions are strong enough to preclude any possibility of protein insertion^19^. Therefore, a high value of π_c_ indicates a high avidity of the protein for the lipid membrane. A reference value is 30 mN.m^−1^, i.e. the surface pressure measured for a natural plasma membrane^22^. As shown in Fig. 2D, the F-452/A mutant induced a significant drop in π_c_ (32.5 mN.m^−1^ compared with 42.5 mN/m^−1^ for the wild-type). In contrast, the homologous F452W mutation did not modify the π_c_ value, in agreement with the kinetics data (Fig. 1C). Overall, these data definitively establish CARC as a functional cholesterol-binding motif. A comparison of these data with previously published values of π_c_ for CRAC-cholesterol interactions^23^ indicates that CARC is in no way less effective than CRAC in sustaining specific cholesterol-binding. The present results are a clear indication that the CARC algorithm should not be overlooked when searching for cholesterol-binding motifs in membrane proteins.

**Figure 3:**
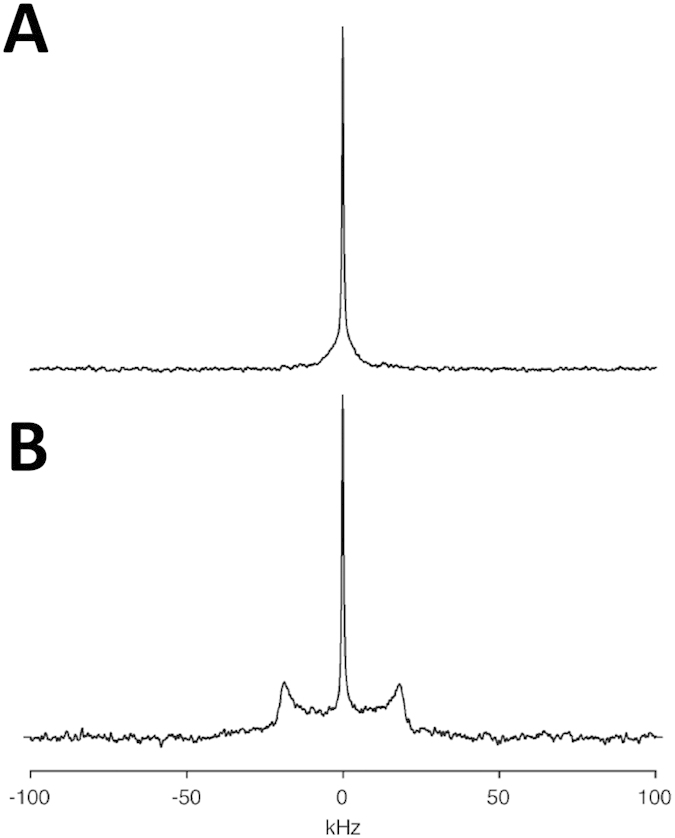
Deuterium NMR spectrum of the AChR γ-TM4 labeled at Ala471 reconstituted into DMPC (**A**) and DMPC/cholesterol (30 mole per cent) (**B**). Spectra recorded at 30 °C and processed as described in the text.

To assess how cholesterol interacts with the Torpedo γ–TM4, a peptide corresponding to Asp_464_-Val_492_ of the nAChR γ-subunit was synthesized with the side chain of deuterated Ala471. Deuterium NMR spectra of the peptide reconstituted into bilayers in the presence and absence of cholesterol were subsequently recorded. The spectra of the lyophilized peptide gave rise to a Pake pattern, characterized by a splitting of 38 kHz as previously observed^24^ (data not shown). Reconstitution of the γTM4 into DMPC vesicles resulted in the deuterium spectrum collapsing into a single resonance (Fig. 3A), with a narrow component arising from residual HDO within the sample superimposed upon a broader component with a width at half height of approximately 8 kHz. In contrast, reconstitution of the γTM4 into DMPC vesicles containing 30 mole per cent cholesterol resulted in a Pake pattern characterized by a splitting of 36.8 kHz upon which a narrow resonance arising from residual HDO is superimposed (Fig. 3B). The 38 kHz splitting observed for the lyophilized material is consistent with a deuterated methyl group undergoing rapid rotation around the C-CD_3_ bond resulting in a scaling of the quadrupolar interaction from its static value of 167 kHz^25^. Similarly, the 36.8 kHz Pake pattern present in the spectrum of γTM4 reconstituted into the DMPC/cholesterol vesicles (Fig. 3B) indicates that, as expected at this temperature, the methyl group still experiences motional averaging of the quadrupolar interaction through rotation around the C-CD_3_ bond, with a further small reduction arising through rapid fluctuations within the bilayer. In contrast, when the γTM4 was reconstituted into pure DMPC bilayers, the deuterium spectra showed that significant motional averaging of the quadrupolar interaction occurred (Fig. 3A).

As described previously^24^, the deuterium spectrum of transmembrane peptides and the M4-TMD in particular are sensitive to rotation around the helical long axis and the membrane normal. The quadrupolar interaction is scaled according to ½(3cos^2^β − 1) where β is the angle that defines the orientation of the tensor with respect to the axis of motional averaging^26^. The extensive averaging of the quadrupolar interaction when the γTM4 is reconstituted into DMPC is consistent with that predicted for an α-helical structure undergoing rotation around the helix long axis, on a timescale that is intermediate to fast with respect to the size of the interaction (microseconds). Similar differences in motional scaling of the quadrupolar interaction were reported previously in transitioning from the gel to liquid crystalline phase, when changes in mobility were attributed to changes in the overall dynamics within the bilayer^24^.

The reduction in mobility may be attributed to a number of factors including perturbation of the phase diagram and/or the formation of larger oligomeric complexes. At the temperature chosen, 30 °C, the DMPC/cholesterol bilayers are expected to be in the liquid-crystalline phase, and indeed no further averaging of the quadrupolar lineshape was observed upon increasing the temperature to 40 °C (data not shown). This suggests that the reduction in mobility arises through the formation of larger complexes within the bilayer with a longer rotational correlation time. Although the binding of cholesterol to the CARC motif present within the γ–TM4 is tight, it appears unlikely that this suffices to generate a sufficient increase in the size of the complex to bring about the reduction in mobility observed. This leads us to propose that the reduction in mobility arises from the cholesterol-induced oligomerization of γ–TM4, which is consistent with previous fluorescence studies showing the oligomerization of γ–TM4 in POPC bilayers in the presence of cholesterol^27^.

The in-silico studies reveal that cholesterol binds to the CARC motif within the γ–TM4 via the β-face (Fig. 1), leaving the α-face exposed. Within model membranes, it has been demonstrated that favorable van der Waals interactions can stabilize cholesterol dimers, either through tail-to-tail or face-to-face interactions. This would suggest that cholesterol-mediated oligomerization is mediated by cholesterol/cholesterol interactions, such as those proposed by Fantini and Barrantes^9^, rather than specific protein/protein contacts for which obvious motifs are absent.

### Disclosure of an overlooked CARC motif in the mitochondrial translocator protein TSPO

**Figure 4:**
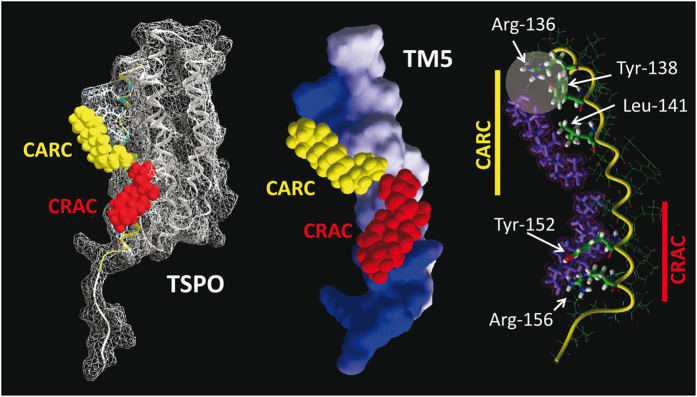
The transmembrane domain of the translocator protein TSPO displays both a CRAC and a CRAC domain. The model on the left represents the docking of cholesterol on the CARC motif (cholesterol in yellow) and on the CRAC motif (cholesterol in red). The 3D structure of cholesterol has been retrieved from PDB entry 1MT5. The model in the middle represents the 5th transmembrane domain (TM5) of TSPO with cholesterol bound to both CARC and CRAC motifs. The model on the right shows the molecular interactions between cholesterol (in purple) and each cholesterol-binding motif of TM5. The hydrogen bond network stabilizing the cholesterol-CARC interaction is indicated by a disk.

The mitochondrial translocator protein TSPO is a perfect example of a biased detection of cholesterol-binding motifs in a transmembrane domain. As noted by Jaremko *et al*. in a recent study^28^, cholesterol binds to TSPO with nanomolar affinity^29^, indicating that the binding domain of this protein is particularly efficient. TSPO, initially characterized as a peripheral benzodiazepine receptor^30^, contains a CRAC domain (residues 149–156) at the C terminus of TM5^13^,^28^. Jaremko *et al*. observed that the 3D topology of certain amino acid side chains in this CRAC domain (Tyr-152, Tyr-153, and Arg-156) was consistent with their interaction with cholesterol. Applying molecular dynamics simulations to the TSPO high-resolution structure published by Jaremko *et al*., i.e. PDB entry 2MGY^28^, we were able to confirm that a cholesterol molecule can bind to the CRAC domain, with an energy of interaction of −44.4 kJ.mol^−1^ (Fig. 4). Moreover, our modeling exercise revealed that both Tyr-152 and Arg-156 might directly interact with cholesterol, in line with the findings of Jaremko *et al*.^28^. However, in addition to CRAC, we found that TSPO also contains a CARC domain, located between amino acid residues 135 and 146, which was not reported in the study by Jaremko *et al*.^28^. Our modeling studies clearly indicate that this domain is not only able to bind cholesterol but that it does so with an energy of interaction of −62.4 kJ.mol^−1^, i.e. significantly higher than the calculated energy of interaction between cholesterol and the CRAC domain (Fig. 4). Moreover, the CARC domain is defined by a triad of essential amino acid residues: Leu/Val, Tyr, and Lys/Arg. We further found that all elements of this triad are involved in cholesterol binding, their contribution to the total energy of interaction being as high as 53%. Finally, the CARC-cholesterol interaction is stabilized by a network of hydrogen bonds linking the −OH group of cholesterol, Arg-135, and Tyr-138 (Fig. 4). A salient feature of our modeling study, overlooked in the work by Jaremko *et al*.^28^, is therefore that the same transmembrane domain of TSPO (TM5) is found to contain both the CRAC and CARC motifs, such that two cholesterol molecules can simultaneously bind the TSPO protein, one in each leaflet of the membrane bilayer, in a symmetrical topology (Fig. 4). This is consistent with the tail-to-tail arrangement of cholesterol in lipid bilayers^9^,^31^,^32^. A similar occurrence of two cholesterol binding motifs (CRAC and CARC) in the same TM domain has been previously reported for the 5th transmembrane domain of the human type 3 somatostatin receptor^9^, which possesses a CRAC domain in the cytoplasmic leaflet (−44 kJ.mol^−1^) and a CARC domain in the exofacial leaflet (−54 kJ.mol^−1^).

**Figure 5:**
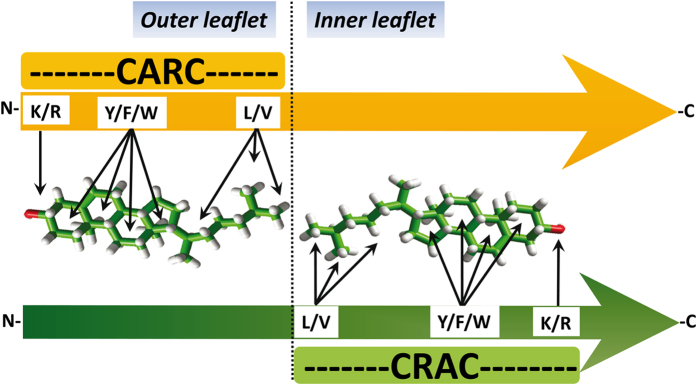
CARC and CRAC are vectorial algorithms. For proteins whose N-terminus is extracellular, the CARC domain is located in the outer leaflet and the CRAC domain is in the inner one. This topology applies for type-1 membrane proteins as well as for TM domains 1, 3, 5, and 7 of G-protein coupled receptors (GPCRs) with seven transmembrane domains. For type-2 membrane proteins (extracellular C-terminus) and domains 2, 4, and 6 of GPCRs, the algorithms still work but in this case CARC is located in the inner leaflet and CRAC in the outer one.

We would like to underscore the fact that both the CRAC and CARC domains are vectorial (“apolar” Leu/Val → “basic” Lys/Arg for CRAC and “basic” Lys/Arg → “apolar” Leu/Val for CARC, from the N-terminus to the C-terminus sequence) (Fig. 5). Thus, only the simultaneous presence of a CARC and a CRAC motif, one in each leaflet of the membrane, can accommodate two opposite (tail-to-tail) cholesterol molecules onto the same transmembrane domain. This ensures that the polar amino acid residues of the motif (Lys/Arg) face the intra- and extracellular milieu, whereas the apolar ends of the motifs (Leu/Val) are deeply buried in the most hydrophobic region of the lipid bilayer. We believe that this new information, confirming and significantly extending the study of Jaremko *et al*.^28^, will be of interest for determining how TSPO interacts with cholesterol in the actual biomembrane environment, and will help understand the mechanisms by which the 18KDa translocator protein mediates the import of cholesterol into the mitochondrion. Jaremko *et al*.^28^ speculated that cholesterol binding at the outside of the TSPO structure, together with the ability of cholesterol to dimerize, could modulate the oligomeric state of the transporter. Our finding of an additional cholesterol site on the same membrane-facing surface of TM provides further energetic grounds to the cholesterol-mediated oligomerization hypothesis.

### How cholesterol interacts with a TM domain displaying both CARC and CRAC motifs

**Figure 6:**
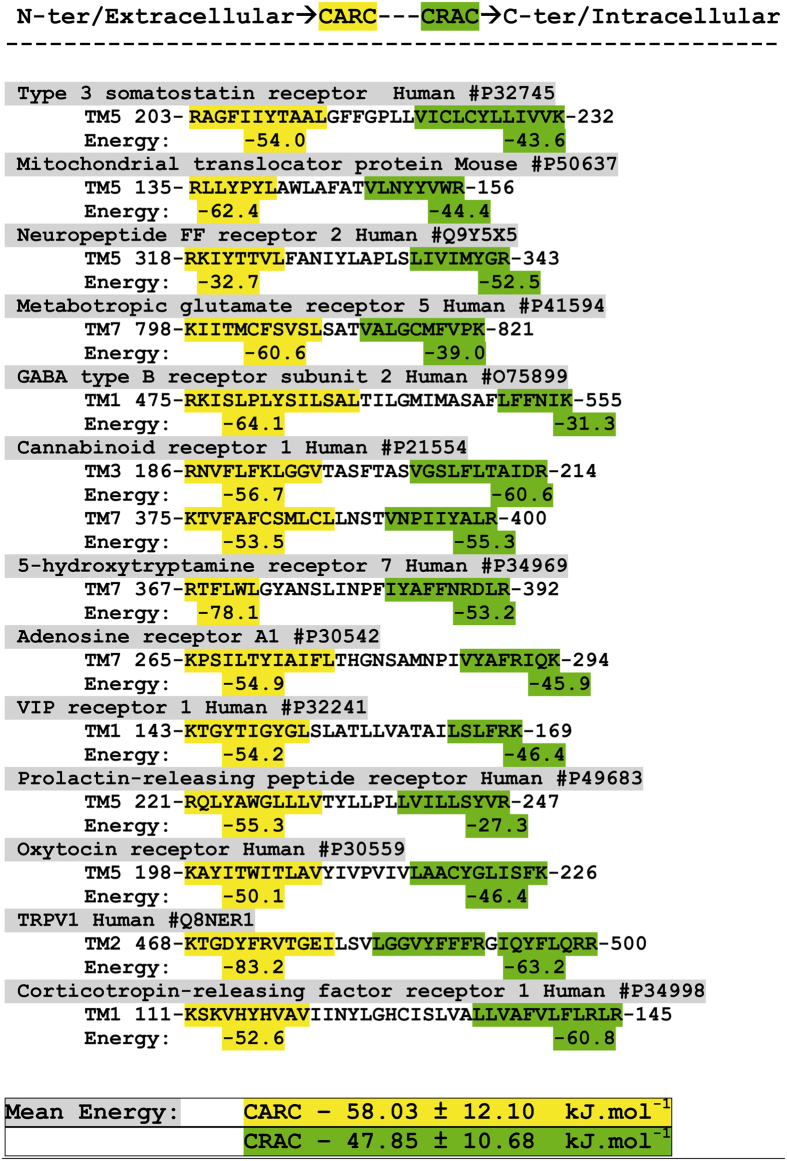
Energetics of cholesterol binding to TM domains displaying both CARC and CRAC motifs. The CARC motif is framed in yellow, and the CRAC motif in green. The calculated energy of interaction (in kJ.mol^−1^) is indicated under each motif. The UniProt entry is indicated for each protein after the # symbol.

**Figure 7:**
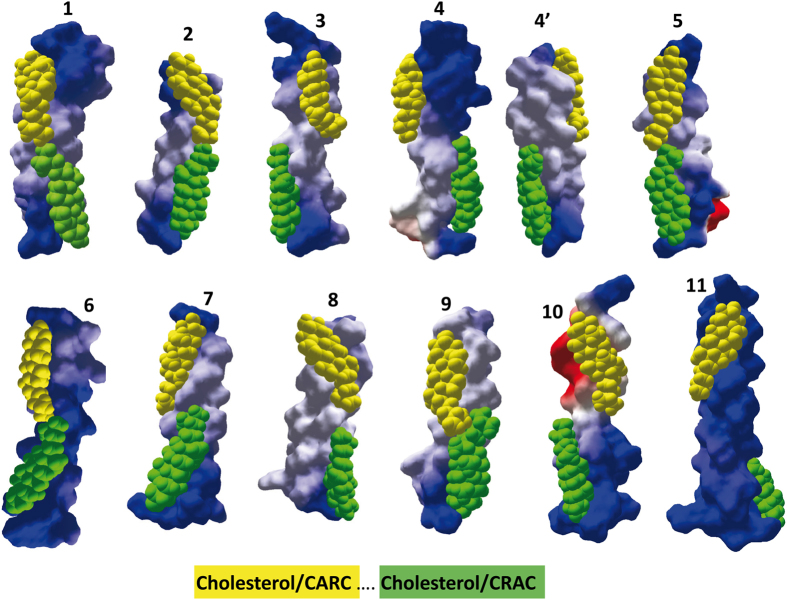
Molecular modeling simulations of cholesterol binding to TM domains displaying both a CARC and a CRAC domain. All TM domains are oriented with the N-terminus upside. Cholesterol bound to CARC (outer leaflet) is in yellow, whereas cholesterol bound to CRAC (inner leaflet) is in green. The energy of interaction of all these complexes is given in Fig. 5. 1) neuropeptide FF receptor (TM5); 2) metabotropic glutamate receptor 5 (TM7); 3) GABA type B receptor subunit 2 (TM1); 4) CB1 receptor (TM3); 4′) CB1 receptor (TM7); 5) 5-HT7 receptor (TM7); 6) adenosine receptor A1 (TM7); 7) VIP receptor 1 (TM1); 8) prolactin-releasing peptide receptor (TM5); 9) oxytocin receptor (TM5); 10) TRVP1 receptor (TM2); 11) corticotrophin-releasing factor receptor 1 (TM1). The UniProt entry of these human receptors is indicated in Fig. 6. Standard colors are used for the electrostatic potential surface rendering of TM domains: positive in blue, negative in red and neutral in white.

Beyond the specific case of the TSPO protein, our data suggest that the simultaneous presence of both CARC and CARC motifs in the same TM domain could imply an interaction with two symmetric cholesterol molecules. To further assess this point, we have examined protein sequence databases for identifying such TM domains and evaluated their possible cholesterol-binding capability. Since CARC and CRAC are vectorial algorithms, we have restricted our study to type I membrane proteins and to domains 1, 3, 5, and 7 of G-protein coupled receptors. Indeed, in *all* these cases, CARC appears first in the amino acid sequence, and is thus located in the outer leaflet. CRAC is found in the inner leaflet. The results of this series of studies are summarized in Figs 6 and 7. In addition to TSPO, our set comprises various signaling membrane receptors including somatostatin, GABA, serotonin, adenosine, VIP and cannabinoid receptors, as well as the voltage-dependent TRVP1 channel. Overall, the mean energy of interaction was −58.03 ± 12.1 kJ.mol^−1^ for CARC and −47.85 ± 10.68 kJ.mol^−1^ for CRAC.

These data indicate that a CARC domain generally exhibits more affinity for cholesterol than a CRAC domain, confirming previous studies performed with smaller protein samples^8^,^9^. Nevertheless, there were some exceptions to this rule (e.g. for neuropeptide FF and corticotropin-releasing factor receptors, as shown in Fig. 6). Molecular dynamics simulations shed some light on the manner in which a TM domain may accommodate two symmetric cholesterol molecules (Fig. 7). Overall one can see that each cholesterol–binding motif lies in one membrane leaflet, with very little overlapping. The only exception is the oxytocin receptor TM5, where a significant interaction between cholesterol molecules is observed. In fact, there are only a limited number of possibilities of symmetric cholesterol interactions, all of which are clearly illustrated in Fig. 7. In a first topology, both cholesterol molecules are placed symmetrically along the same axis, so that they can interact through the terminal methyl groups of their iso-octyl chain. This is typically the case for neuropeptide FF receptor, metabotropic glutamate receptor 5, the serotonin 5-HT7 receptor, or the adenosine receptor A1. In the case of the oxytocin receptor, the interaction between cholesterol molecules involved both the iso-octyl chain (CARC cholesterol, in yellow) and the D ring of the sterane backbone (CRAC cholesterol, in green), resulting in a tighter association of cholesterol molecules.

In a second topology, the cholesterol molecules are slightly shifted, and they are still entirely visible in the same view, but can no longer physically interact. This is the case for VIP, prolactin, and GABA type B receptors. In a third topology the two cholesterol molecules are located on opposite faces of the TM helix, as is the case for TM3 and TM7 in the CB1 receptor. Incidentally, this particular geometry may allow a close contact between the two TM domains through a combination of cholesterol-cholesterol and protein-protein interactions. In the case of CB1, it is interesting to note the very good shape complementarity of TM3 and TM7 in the outer leaflet region, and the possibility of a face-to-face association of the cholesterol molecules bound to the CRAC motifs in the inner leaflet (cholesterol in green). The TRVP1 receptor channel also belongs to this topological family. In this case, the cholesterol molecules are wrapped around the TM helix, in a typical “DNA-like” configuration. A similar cholesterol wrapping occurred for the corticotrophin-releasing factor 1, which accommodates tilted conformers of cholesterol in each membrane leaflet. In order to determine whether both CARC and CRAC domains from the same TM domain can actually bind cholesterol, we selected the 7^th^ TM domain of the human serotonin 5-HT7 receptor which has a CARC motif in the exofacial leaflet and a CRAC motif in the opposite leaflet.

**Figure 8:**
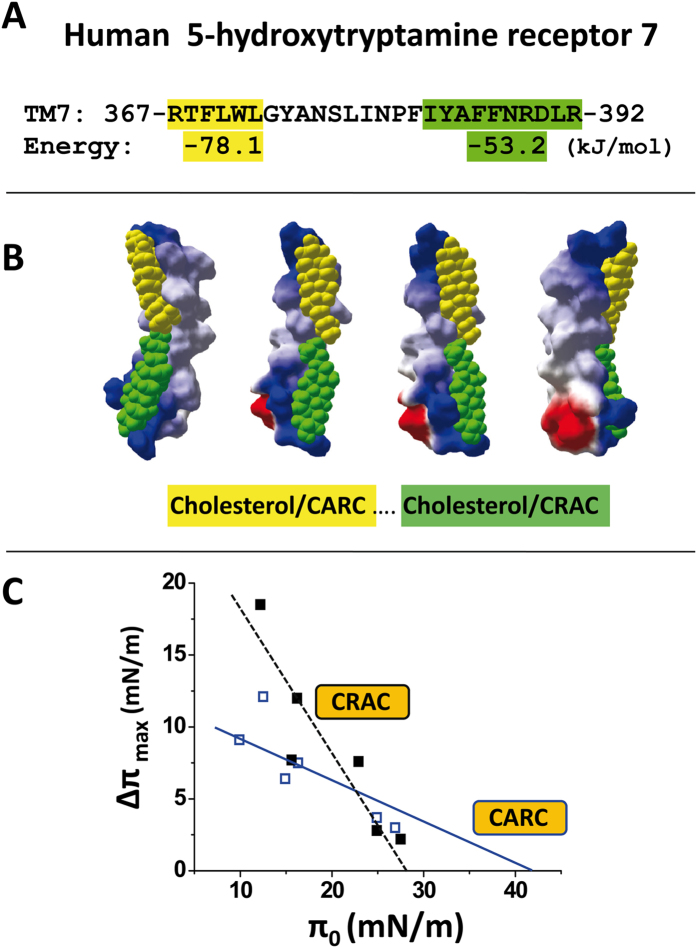
Cholesterol-binding properties of CARC and CRAC domains derived from the same TM domain. (**A**) The 7th TM domain of the human serotonin 5-HT7 receptor displays a CARC motif (yellow) in the exofacial leaflet and a CRAC motif (green) in the cytoplasmic leaflet. The energy of interaction between each motif and cholesterol is indicated under the amino acid sequence. (**B**) Distinct views of the TM domain with cholesterol in yellow bound to CARC and cholesterol in green bound to CRAC. (**C**) Interaction of the CARC and CRAC peptides derived from the 7th TM domain of the human serotonin 5-HT7 receptor with cholesterol monolayers. Standard colors are used for the electrostatic potential surface rendering of TM domains: positive in blue, negative in red and neutral in white.

Two synthetic peptides containing either the CARC or the CRAC motifs were tested for their cholesterol binding capability (Fig. 8): **RTFLWL**GYANSL for CARC and SLINPF**IYAFFNRDLR** for CRAC (Fig. 8A). Molecular dynamics simulations indicated that CARC displayed a higher affinity for cholesterol than CRAC (−78.1 and −53.2 kJ/mol for the CARC/cholesterol and the CRAC/cholesterol complexes, respectively). However, both motifs appeared remarkably complementary for cholesterol as illustrated in the molecular models of Fig. 8B. Thus, it was especially interesting to determine whether the higher affinity of CARC vs. CRAC for cholesterol, as predicted in silico, could be experimentally confirmed. To this end, we prepared a series of cholesterol monolayers at various initial pressure (π_0_) values and studied the insertion of the CARC and CRAC peptides derived from the 7^th^ TM domain of the human serotonin 5-HT7 receptor (Fig. 8C). When added underneath these cholesterol monolayers, both peptides induced a marked increase in the surface pressure. However, the CRAC/cholesterol interaction was no longer detected above the critical pressure of insertion (π_c_) of 28 mN.m^−1^. In contrast, the CARC peptide remained active until reaching a π_c_ value of 42 mN.m^−1^. Since a higher value of π_c_ indicates a higher avidity of the peptide for the reconstituted lipid membrane, these physicochemical experiments demonstrated that CARC has a higher avidity for a cholesterol-containing membrane than CRAC. Overall, these data fit quite remarkably with our in silico studies.

## Discussion

In this study we present a thorough characterization of the functionality of the CARC motif as a high affinity cholesterol-binding domain for membrane-bound receptors. Though we had already carried out a series of careful in silico studies that extensively described the molecular mechanisms controlling CARC-cholesterol interactions in several distinct proteins^8^,^9^, we had not yet established whether cholesterol could physically interact with a CARC domain. We have now subjected this to experimental test by studying the interaction of a prototype CARC domain with cholesterol monolayers. Our strategy for selecting such a representative CARC domain was based on photolabeling studies which identified Asp-448 as the main target for [^3^H]Azicholesterol in the 4^th^ TM domain of the *Torpedo* AChR γ–subunit^18^. Since this residue is contiguous to a CARC domain, we decided to challenge our molecular dynamic simulation methods with this result. Thus we studied the interaction of *Torpedo* γ–TM4 with Azicholesterol. We found that this cholesterol derivative interacted with the amino acid residues defined by the CARC algorithm, namely Lys-449, Phe-452 and Leu-456. Moreover, the lateral Azi group was at a distance of only 2 Å from the side chain of Asp-448, consistent with a UV- reaction. This result fully validated our modeling approach. Therefore, we modeled the interaction between cholesterol and either wild-type or mutant TM4 peptides. The wild-type CARC motif displayed a high affinity for cholesterol. All three amino acids defining the CARC domain were found to interact with cholesterol, especially the central Phe-452 residue. Interestingly, replacing this aromatic residue with alanine (F-452/A mutant) resulted in a significant loss of affinity. This important result was fully confirmed by physicochemical studies of CARC-cholesterol interactions. Moreover, the more conservative F-452/W mutation had no effect, indicating that it is the aromatic nature of Phe-452, and not its specific structure, that is required for optimal binding. This is in line with previous studies suggesting that CARC motifs could contain any of the three aromatic residues, i.e. Phe, Trp or Tyr^8^,^9^. The other major outcomes of our physicochemical studies are the demonstration of lipid-specificity (CARC recognized cholesterol but not phosphatidylcholine) and the concentration dependency of the binding (saturation was reached for peptide concentrations <10 μM). From the deuterium NMR studies conducted, it is clear that the presence of cholesterol within the bilayer results in a reduction in the rotational motion of the peptide within the bilayer, a change consistent with cholesterol promoting the oligomerization of *Torpedo* γ–TM4. The functional significance of this in the intact receptor remains to be elucidated, but the location of the CARC domain on the lipid-facing surface of the helix may play a role in cholesterol-mediated clustering of the receptor.

Overall, our findings show that CARC is a functional cholesterol-binding motif as efficient as CRAC.

Historically, CRAC was the first cholesterol-recognition motif ever characterized^13^. This may explain why it remains the most popular and sometimes the exclusive algorithm considered for identifying cholesterol-binding motifs in membrane proteins^28^. However, problems arose when membrane proteins known to interact with cholesterol were shown not to display any CRAC motifs in their membrane-spanning regions. Such was the case of the AChR, and this deficiency led to the finding of a reversed version of the CRAC algorithm, that we logically referred to as “CARC”^8^. Molecular dynamics simulations indicated that the CARC motifs identified in the TM domains of a broad range of membrane receptors could bind cholesterol with high affinity. Another interesting feature of CARC is that the consensus sequence starts with a basic residue (Arg or Lys) so that a CARC motif is ideally suited for interaction with cholesterol in the outer leaflet of biological membranes. Indeed, the N-terminal domain of type I membrane proteins is extracellular, such that the carbon chain enters the membrane in the N- to C-terminus direction (Fig. 5). This is also the case for TM domains 1, 3, 5, and 7 of G-protein-coupled receptors. Moreover, the presence of a CARC domain in the outer leaflet is still consistent with the occurrence of a symmetric CRAC domain in the inner leaflet. Since the consensus CRAC sequence starts with an aliphatic residue (Leu or Val), its N-terminal part is expected to interact with the apolar groups of cholesterol (sterane, methyl and iso-octyl) in the inner leaflet (Fig. 5). In other words, the sequential chaining of CARC and CRAC motifs in the amino acid sequence of a TM domain, starting from the N-terminus, is consistent with the binding of a cholesterol molecule in each leaflet. To investigate this interesting possibility, we selected a set of TM domains belonging to a broad range of signaling receptors and displaying this vectorial [CARC---CRAC] pair of cholesterol-binding motifs. Molecular dynamics simulations of the whole TM domain were conducted to assess: i) whether two cholesterol molecules could be actually docked onto these domains, and ii) to determine to which extent the CARC and CRAC motifs were involved in cholesterol binding (Figs 6, 7, 8). The results of this large-scale modeling approach indicate that in all cases, two cholesterol molecules could be simultaneously docked on the TM domain, one in each leaflet. There was no indication of any steric hindrance preventing the binding of the second cholesterol once the first one was bound. Moreover, physicochemical studies with cholesterol monolayers confirmed that both the CARC and CRAC domains of the 7^th^ TM domain of the human serotonin 5-HT7 receptor have cholesterol-binding properties (Fig. 8). Overall, cholesterol was found to interact with the CARC motif in the outer leaflet, and with CRAC in the inner leaflet. The three main amino acid residues defining the CARC and CRAC motifs were always involved in the interaction. Nevertheless, it should be mentioned that for both CARC and CRAC, the central aromatic residue could be either Phe or Tyr (and even Trp in the case of CARC). This finding is consistent with the nature of the interaction between cholesterol and aromatic rings, i.e. the CH-π stacking interaction^33^. In this respect, one should also note that branched aliphatic residues (Leu/Val/Ile) are perfectly suited to accommodate the methyl “spikes” of cholesterol. In the same way, the terminal basic residue of the motif often forms a hydrogen bond with the oxygen atom of the OH group of cholesterol^9^. Thus, the selection of CARC and CRAC as cholesterol-binding motifs is justified by robust physicochemical rules. These rules can be put into practice via a combination of London, CH-π stacking and hydrogen bonding that cooperate to control protein-cholesterol interactions in the membrane environment^9^.

### Conclusion: guidelines for the prediction of cholesterol-binding motifs in TM domains

The data reported in the present study strongly suggest that the dual presence of CARC and CRAC motifs within the same TM domain reflects a mirror code controlling protein-cholesterol interactions in both the outer and inner membrane leaflets. Deciphering this code allowed us to elaborate straightforward guidelines for the accurate detection of cholesterol-binding motifs in membrane proteins. These guidelines are detailed below and illustrated in the Supplementary Information.

The strategy is based on examination of the amino acid sequence of a protein as downloaded from any sequence database. As an example we will deal with the case of the human CB1 receptor (UniProt entry #P21554). This receptor belongs to the family of GPCRs and thus contains seven TM domains. The location of these domains is generally indicated in the database. In this case, the seven TM domains are defined as segments 117–142 (TM1), 155–175 (TM2), 188–212 (TM3), 233–255 (TM4), 274–299 (TM5), 345–365 (TM6), and 378–399 (TM7). Since the N-terminus of this receptor is extracellular, the typical [CARC---CRAC] series (from the N- to the C-terminus) may occur in TM1, TM3, TM5, and TM7. The first step is to analyze these TM domains and then proceed to see what to do with the others. Since TM1 does not contain any aromatic residue close to the endings, one can ignore it and continue on to TM3. This sequence is interesting because it contains Phe residues at both endings (Supplementary Fig. S1). One must be aware that charged residues lining TM domains are often not included in the TM sequence, although they might well be located at the apolar-polar interface of the membrane^17^. Thus one needs to check for the presence of Lys or Arg residues not only inside the TM domains but also nearby. This is indeed the case for TM3, which does not contain any basic residue but displays Arg residues just upstream from the N-terminus and downstream from the C-terminus (Supplementary Fig. S1). Thus, we can now select the sequence of an “extended” TM3 domain corresponding to 186–214 instead of the “database-predicted” 188–212 fragment. Since we have identified two of the three CARC/CRAC hallmark residues at each ending, it remains to be seen whether a Leu or Val residue can complete these motifs. One can easily see that this is indeed the case: there is a CARC domain at the N-terminus (186-**R**NV**F**L **F**K**L**GG**V**-196) and a “mirror” CRAC domain at the C-terminus (204-**V**GSL**F**LTAID**R**-214). Note that there are in fact several overlapping CARC motifs at the N-terminus of TM3, due to the presence of two Phe residues and both a Leu and Val residue in the C-terminal region. In this case, the largest possible sequence is selected. In TM5, we can find a CRAC sequence in the inner leaflet (591-**V**YA**Y**M**Y**ILW**K**-300), but no CARC motif in the outer leaflet. In TM7, both CARC and CRAC motifs could be detected (Supplementary Fig. S1). Thus, a rapid survey of the amino acid sequence of the human CB1 receptor enables one to identify two TM domains displaying both CARC and CRAC motifs. For the three remaining TM domains (i.e. TM2, TM4, and TM6), the N-terminus comes from the cytosol. In this case, we can still look for the typical [CARC---CRAC] couple, yet we have to keep in mind that in this case CARC will be located in the inner leaflet and CRAC in the outer one. Otherwise, the same sequence analysis procedure can be carried out on these domains too. TM2 has Phe residues but lacks basic amino acid residues. TM4 displays a CARC domain (232-**K**AVVA**F**C**L**-239), but no CRAC motif. Finally, TM6 has neither a CARC nor a CRAC motif. In summary, we found the typical [CARC---CRAC] mirror duet in TM3 and TM7, a single CRAC domain in the inner leaflet region of TM5 and a single CARC domain in TM4 (also in the inner leaflet region). These guidelines can be applied for the identification of cholesterol-recognition motifs in either the outer or inner leaflet-spanning region of any TM domain. Beyond the specific case of the proteins studied here, we would like to emphasize the need to search for both CRAC and CARC motifs in transmembrane protein domains which, in more cases than one would expect, interact with two symmetric cholesterol molecules.

### Perspectives

Future studies will be necessary to assess all biological and clinical consequences of the mirror code. One important point concerns the status of cholesterol-binding domains in yeasts. This is an interesting issue because yeasts, which express ergosterol instead of cholesterol, do not always sustain the functional expression of human receptors^34^. For instance, ligand binding to μ-opioid receptor was increased in transfected yeast cells when ergosterol was replaced by cholesterol^35^. These findings suggest that human CARC/CRAC domains might be specific for cholesterol and cannot mediate functional ergosterol binding. This sterol specificity could be due to subtle conformational differences between cholesterol and ergosterol, the former being more flexible and the latter more rigid due to the presence of several extra-double bonds^36^,^37^.

Another potential application of the mirror code concerns ABC transporters involved in cholesterol transport across the plasma membrane. Both CARC and CRAC motifs have been identified in these proteins^38^,^39^,^40^. However, the simultaneous presence of both CARC and CRAC within the same TM domain has not been investigated as yet. Interestingly, the TM8 domain of ABCG2 and ABCG5 displays a dual CARC/CRAC motif (Supplementary Fig. S2). Moreover, we detected a similar pair at each ending of the TM8 domain of multidrug resistance protein 1A^41^ (Supplementary Fig. S3). Taken together, these findings raise the interesting possibility that such distant CARC/CRAC pairs could favor the translocation of cholesterol along the TM domain.

Apart from cholesterol translocation, the simultaneous presence of CARC and CRAC motifs in a TM domain might reinforce the interaction of the protein with cholesterol-rich plasma membrane domains. In this respect, we should now consider three categories of non-annular binding sites for cholesterol: in the outer leaflet, in the inner leaflet, or in both leaflets. The mirror code described herein applies to the latter case. All the proteins displaying this intriguing feature are probably involved in a functional interaction with cholesterol in both leaflets of the plasma membrane and we anticipate that there will not be many exceptions to this rule. More generally, the mere presence of a CARC or a CRAC motif in a TM domain implies that the protein has the intrinsic capability to interact with cholesterol, provided that cholesterol molecules are accessible in the membrane area of the protein. Finally, cholesterol has been shown to interact with tilted peptides that do not fulfill the CARC/CRAC algorithms^42^, yet this feature is generally a characteristic of infectious and amyloid proteins^9^,^23^.

At this stage, the potential applications of the mirror code in the clinical realm remain unclear. Since CARC/CRAC motifs are present in many membrane proteins, any cholesterol modulator might cause some unpredictable effects, this perhaps being a disadvantage in drug target design and clinical treatment: nevertheless, disrupting the association of membrane cholesterol with amyloid proteins efficiently prevented amyloid pore formation in cultured brain cells without undesirable side effects^43^,^44^. Moreover, mutations of basic residues in TM domains are often associated with protein malfunction and subsequent disease in humans^45^. Since basic residues are present in both CARC and CRAC domains, it would be interesting to know whether an alteration of cholesterol binding, responsible for protein instability, might be involved in the pathophysiology of these diseases.

## Methods

### Molecular modeling/docking

The amino acid sequence of the TM domains was retrieved from the UniProtKB database (http://www.uniprot.org). Each TM α-helix was generated with the Hyperchem software (ChemCAD, Obernay, France), after which geometry optimization was achieved with the unconstrained optimization rendered by the Polak-Ribière conjugate gradient algorithm. Molecular dynamics simulations of each TM domain in the presence of cholesterol were then performed for iterative periods of 1 ns in vacuo with the Bio + (CHARMM) force field^46^ as described previously^19^,^43^,^47^,^48^. The energy of interaction of CARC-cholesterol and CRAC-cholesterol complexes was estimated with the ligand energy inspector function of Molegro Molecular Viewer^49^. Molecular structures were visualized with Hyperchem, Molegro Molecular Viewer or Swiss-PDB viewer^50^.

### Monolayer studies

All synthetic peptides (purity > 95%) were obtained from Schafer-N, Copenhagen, Denmark. Lipids were purchased from Matreya (Pleasant Gap, PA, USA). Ultrapure apyrogenic water was from Biorad (Marnes La Coquette, France). Peptide-cholesterol interactions were studied with the Langmuir film balance technique^19^ using a Kibron microtensiometer (μTROUGH SX, Kibron Inc. Helsinki, Finland) as previously described^23^. Unlike in graphite, where cholesterol molecules are regularly arranged in pairs oriented head-to-head^51^, the aqueous subphase allows the formation of a compressible monolayer^52^ with the polar head group oriented towards water^19^. All experiments were carried out in a controlled atmosphere at 20 ± 1°C. Monomolecular films of the indicated lipid were spread on pure water subphases (volume of 800 μl) from hexane:chloroform:ethanol (11:5:4, by vol.). After spreading of the film, 5 min was allowed for solvent evaporation. The synthetic peptide was injected in the aqueous subphase with a 10-μl Hamilton syringe, and pressure increases were continuously recorded as a function of time. The data were analyzed with the FilmWareX 3.57 program (Kibron Inc.). The accuracy of the system under our experimental conditions was ± 0.25 mN.m^−1^ for surface pressure. When injected into water (i.e. in absence of lipid monolayer), the synthetic peptides did not affect the superficial tension of water. Hence, any surface increase induced by the peptide injected underneath a lipid monolayer indicated a physical interaction between the peptide and the lipid monolayer.

### Sample preparation for NMR Spectroscopy

A peptide composed of N-Asp-Lys-Ala-Cys-Phe-Trp-Ile-(D3)Ala8-Leu-Leu- (1–13C)Leu11-Phe-Ser-Ile-(15N)Gly15-Thr-Leu-Ala-Ile-Phe-Leu-Thr-(2-13C)Gly23-His-Phe-Asn-Gln- Val-C (corresponding to Asp464 to Val492 in the intact γ-subunit) was synthesized using conventional FMOC chemistry by the NSR Centre (Nijmegen, Netherlands) with labeled amino acids purchased from Cambridge Isotopes Ltd (MA, USA). The peptide was reconstituted into bilayer composed of DMPC and DMPC by co-solubilizing ~1mg of peptide with dimyristoyl-phosphatidylcholine (DMPC) at a lipid to protein ratio of 25:1 (L/P). Following solubilization, the solvent was removed under high vacuum overnight resulting in the formation of a thin lipid/protein film. The film was resuspended in deuterium depleted water (Sigma, UK) and 1 mM TCEP, to ensure the peptide remained reduced and monomeric, and subjected to 10 freeze- thaw cycles to produce a homogeneous population of multilamellar vesicles hydrated at 30% (w/v). Samples containing 30 mole per cent cholesterol were prepared in a similar manner with the appropriate mass of cholesterol added prior to co-solubilization to ensure a 30% molar concentration of cholesterol with respect to the phospholipid. Prior to NMR experiments, samples were transferred into a thin-walled 3.2 mm rotor by low-speed centrifugation.

### Deuterium NMR Spectroscopy

Deuterium NMR studies were conducted on an Agilent DD2 600 MHz NMR spectrometer equipped with 3.2 mm MAS triple resonance magic-angle spinning probe, without spinning. Static deuterium NMR spectra were recorded at 92.17 MHz using a standard quadrupolar echo sequence with π/2 pulses of 4 μs and an inter-pulse delay of 50 μs. Temperature was regulated to 30 °C and 128,000 acquisitions were typically acquired. Deuterium spectra were referenced externally to D_2_O. Prior to Fourier transformation the deuterium free induction decay was left-shifted to the top of the echo and 500 Hz line-broadening applied. All NMR data were processed in matNMR^53^.

## Additional Information

**How to cite this article**: Fantini, J. *et al*. A mirror code for protein-cholesterol interactions in the two leaflets of biological membranes. *Sci. Rep*. **6**, 21907; doi: 10.1038/srep21907 (2016).

## Supplementary Material

Supplementary Information
