# Supplementary material for: A mirror code for protein-cholesterol interactions in the two leaflets of biological membranes
Source: Sci Rep. 2016 Feb 26;6:21907. doi: 10.1038/srep21907 (PMC4768152; doi:10.1038/srep21907)
Supplement: Supplementary Information [file srep21907-s1.pdf]

Supplementary Information for :

A mirror code for protein-cholesterol interactions in the inner and outer leaflets of biological membranes

Jacques Fantini, Coralie Di Scala, Luke S. Evans, Philip T.F. Williamson and Francisco J. Barrantes

Figure S1: Guidelines for the prediction of cholesterol-binding motifs in TM domains.

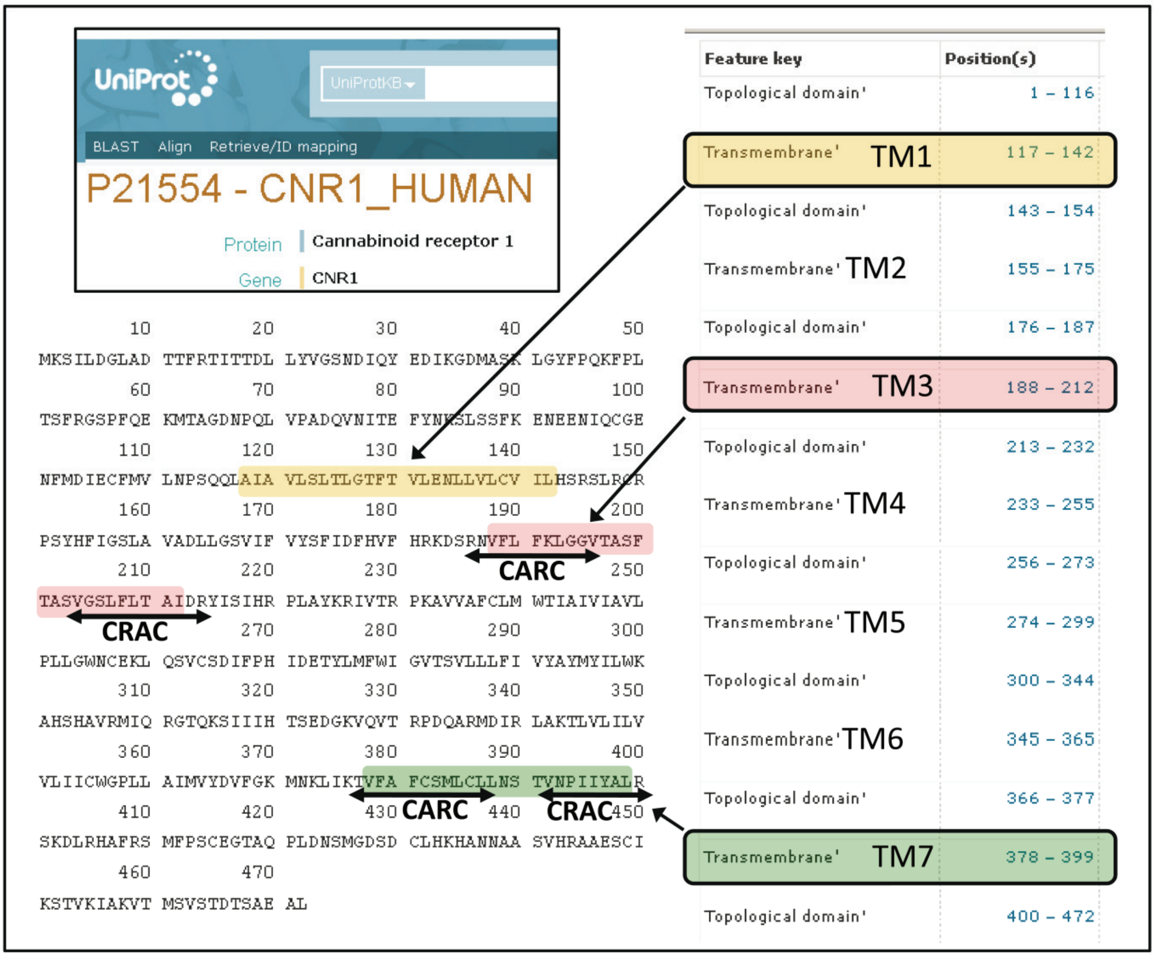

Figure S2: Dual CARC/CRAC motifs in ABC transporters.

**Human ABCG2 TM2:**	**426-RAGVLFFLTTNQCFSSVSAVELFVVEK-452**
**Human ABCG5 TM2:**	**418-RVGLLYQFVGATPYTGMLNAVNLFPVLR-446**

Amino acid sequences were retrieved from Uniprot entries Q9UNQ0 (ABCG2) and Q9H222 (ABCG5). The CARC motif is in yellow, CRAC is in green.

Figure S3: Dual CARC/CRAC motifs in The MDR1A protein.

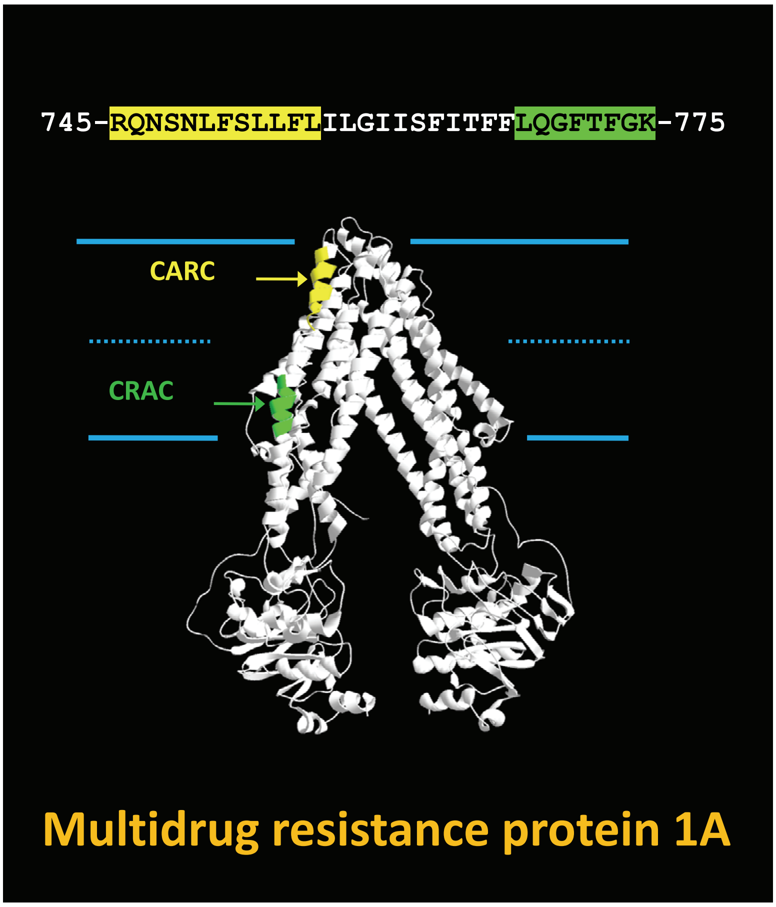

CARC (yellow) and CRAC (green) in the TM8 domain of the ABC transporter MDR1A (Uniprot entry P21447) have been localized in the 3D structure retrieved from PDB entry 3G5U (Aller et al. (2009) *Science* **323**: 1718-1722).
